# Clinical Impact of Germline Pathogenic Variants in High-risk Prostate Cancer Treated with Radiotherapy

**DOI:** 10.1016/j.euros.2026.07.002

**Published:** 2026-07-27

**Authors:** Javier Galego-Carro, Marta Santamariña, Ana Blanco-Pérez, Miguel E. Aguado-Barrera, Jorge Amigo, Olivia Fuentes-Ríos, Carla Coedo-Costa, Patricia Calvo-Crespo, Paula Peleteiro-Higuero, Ana María Carballo-Castro, Begoña Taboada-Valladares, Antonio Gómez-Caamaño, Ana Vega

**Affiliations:** a Instituto de Investigación Sanitaria de Santiago de Compostela (IDIS), Santiago de Compostela, Spain; b Fundación Pública Galega de Medicina Xenómica (FPGMX), Santiago de Compostela, Spain; c Universidad de Santiago de Compostela (USC), Santiago de Compostela, Spain; d Centro de Investigación en Red de Enfermedades Raras (CIBERER), Santiago de Compostela, Spain; e Servicio de Oncología Radioterápica, Complejo Hospitalario Universitario de Santiago (CHUS), Santiago de Compostela, Spain

## Abstract

**Background:**

Pathogenic and likely pathogenic (P/LP) germline variants in cancer susceptibility genes (CSGs) are detected in 5–10% of patients with prostate cancer, but their clinical impact in high-risk disease remains unclear.

**Objective:**

This study aimed to assess the prevalence of P/LP variants in CSGs among patients with high-risk prostate cancer and their association with oncologic outcomes in patients treated with radiotherapy.

**Methods:**

We conducted a retrospective study of 600 men with high-risk prostate cancer. Germline DNA was analyzed using a 19-gene panel, and gene-level variant prevalence was compared with non-cancer control cohorts using Fisher’s exact test. Time-to-event analyses were performed in the 390 patients treated with radiotherapy as primary curative intent. The primary endpoint was overall survival (OS), assessed by Kaplan-Meier and multivariable Cox regression. Secondary endpoints included biochemical recurrence, distant metastasis, prostate cancer–specific mortality (PCSM), and second primary malignancies, analyzed using cumulative incidence functions and Fine-Gray competing-risk models.

**Key findings and limitations:**

P/LP variants were identified in 8.0% of patients (*n* = 48). *CHEK2*, *ATM*, and *BRCA2* variants were significantly enriched in patients compared with non-cancer control cohorts. *BRCA1/BRCA2* carriers had worse OS (10-yr OS rate: 19% vs 57%; *p* = 0.021) and higher cumulative incidence of biochemical recurrence events (10-yr cumulative incidence: 57% vs 28%; *p* = 0.048), distant metastases (57% vs 18%; *p* = 0.004) and PCSM (33% vs 4.3%; *p* = 0.001) compared with non-carriers. *CHEK2* carriers showed heterogeneous family cancer histories and a higher incidence of second primary malignancies (47% vs 15%; *p* = 0.003). *ATM* carriers showed no metastatic progression or PCSM during follow-up. Limitations include the single-institution design and small gene-specific subgroups.

**Conclusions and clinical implications:**

Germline P/LP variants are prevalent in high-risk prostate cancer and define gene-specific subgroups with distinct oncologic outcomes, supporting the role of germline genetic testing in risk stratification and treatment decision-making.

## Take Home Message

About 8% of patients with high-risk prostate cancer harbor pathogenic variants in cancer susceptibility genes, particularly *CHEK2*, *ATM* and *BRCA2*. These carriers exhibit distinct clinical outcomes, supporting the potential of genetic testing to guide personalized screening and treatment strategies.

ADVANCING PRACTICE **What does this study add?**

This study analyzed a large cohort of patients with high-risk prostate cancer, with time-to-event analyses restricted to a subgroup treated with radiotherapy as primary curative treatment. Germline alterations in *BRCA1*, *BRCA2*, *CHEK2*, and *ATM* are associated with distinct clinical trajectories, extending beyond overall survival to metastatic progression and second primary malignancies. These findings contribute to the understanding of the prognostic implications of germline variation in high-risk prostate cancer and support the role of germline testing in risk stratification and long-term management strategies.

**Clinical Relevance**

Germline pathogenic variants, particularly in *BRCA1* and *BRCA2*, may identify a subgroup of patients with high-risk prostate cancer who are at increased risk of recurrence, metastasis, and prostate cancer–specific mortality following radiotherapy. These findings support the potential role of germline genetic testing in refining risk stratification and informing individualized treatment and follow-up strategies. However, the predictive value of gene-specific germline variants requires further validation in larger, prospective cohorts before they can be routinely incorporated into clinical decision-making. Associate Editor: Roderick C.N. van den Bergh, MD PhD.

**Patient Summary**

In this study, we examined whether inherited genetic changes influence outcomes for men with high-risk prostate cancer treated with radiotherapy. We found that about one in 12 men carried an inherited alteration; some were linked to a higher risk of cancer spread or worse survival, others to an increased risk of developing additional cancers, and some to more favorable outcomes. These findings suggest that genetic testing could help doctors better understand long-term risks and tailor follow-up and treatment for each patient.

## 1 Introduction

Prostate cancer represents a growing global health burden and is projected to become one of the most prevalent malignancies worldwide, with incident cases expected to nearly double by 2040, reaching 3 million new diagnoses annually [1]. In the context of this increasing incidence and prevalence, improving strategies for early detection, risk stratification and targeted treatment are needed to optimize patient outcomes and reduce disease-related mortality.

High-risk prostate cancer represents a particularly challenging clinical entity, characterized by a substantial risk of recurrence and metastatic progression despite treatment with curative approaches such as radical prostatectomy or definitive radiotherapy. Although this subgroup accounts for only approximately 20% of newly diagnosed localized and locally advanced cases, it accounts for a disproportionately large share of prostate cancer–related deaths, exceeding 65% [2]. While radical prostatectomy and definitive radiotherapy achieve initial disease control in many patients, a significant proportion still experience disease recurrence or progression to metastatic stages. This variability in clinical outcomes reflects the underlying biological heterogeneity of high-risk disease [3] and underscores the need for improved molecular stratification tools to more accurately identify patients at greatest risk of adverse outcomes.

In recent years, molecular characterization has identified potential biomarkers associated with disease aggressiveness and treatment response [4], [5]. A substantial proportion of patients with prostate cancer harbor germline pathogenic and likely pathogenic (P/LP) variants in DNA repair genes (eg, *BRCA1*, *BRCA2*, *ATM* and others) [6], [7], [8], [9], which may have important implications for clinical decision-making, including informing therapeutic and management strategies, determining eligibility for clinical trials [10], and enabling familial risk assessment and genetic counseling. Despite the increasing adoption of germline testing in metastatic prostate cancer, the prevalence and clinical impact of these variants in patients with high-risk localized or locally advanced disease remain insufficiently characterized. Most available evidence derives from heterogeneous cohorts or predominantly surgically-treated populations, and data specifically addressing outcomes after definitive radiotherapy are limited.

Therefore, we conducted a retrospective analysis of a well-characterized cohort of 600 patients with high-risk prostate cancer. Using a comprehensive panel of cancer susceptibility genes (CSGs), we aimed to determine the prevalence of germline P/LP variants and to explore their associations with clinicopathological characteristics and oncologic outcomes, including survival, metastatic progression, and secondary malignancies. These analyses were conducted in predefined subsets of the cohort according to treatment modality and follow-up availability.

## 2 Methods

### 2.1 Patients and controls

A total of 600 patients with high-risk prostate cancer from the RADIOGEN cohort [11] enrolled between 2005 and 2021 were included according to the European Association of Urology (EAU) criteria for biochemical recurrence (BCR) [12]. High-risk disease was defined as localized prostate cancer with prostate-specific antigen (PSA) >20 ng/ml, Gleason score ≥8, or clinical stage ≥cT2c, or the presence of locally advanced disease. Patients with de novo metastatic prostate cancer were excluded; staging was performed using bone scintigraphy and/or positron emission tomography-computed tomography, according to institutional practice. No patients received poly(ADP-ribose) polymerase (PARP) inhibitor therapy. Clinical characteristics included age, PSA level, Gleason score, Tumor-Node-Metastasis stage, family history of cancer and treatments.

All 600 patients were eligible for descriptive and P/LP prevalence analyses. Time-to-event analyses were restricted to a predefined subgroup of 390 patients treated with external beam radiotherapy as the primary curative treatment modality and with available follow-up data at our institution. Patients were excluded from outcome analyses if they had undergone prior radical prostatectomy (*n* = 146) or if no post-treatment follow-up information was available at the time of data cut-off (*n* = 64). Within the radiotherapy treated subgroup, comorbidities and treatment regimens (including dose, fractionation, pelvic nodal irradiation, and hormone therapy) were heterogeneous (Supplementary Table 1). Median follow-up for men still at risk was 10.4 yr (interquartile range [IQR], 8.9–12.8 yr). No minimum follow-up duration was predefined; however, all patients had at least one post-treatment clinical assessment.

Written informed consent was obtained from all patients, in accordance with the protocol approved by the Galician Ethics Committee for Clinical Research (2016/597), and in compliance with the Declaration of Helsinki.

Two non-cancer control groups were established to identify genes enriched for germline P/LP variants in patients with high-risk prostate cancer: (1) an in-house cohort of 10 577 individuals and (2) an external cohort of 51 285 individuals from gnomAD v2.1.1 (non-Finnish European population).

### 2.2 Germline DNA sequencing

Genomic DNA was extracted from peripheral blood using the *Chemagic DNA Blood 100* kit (PerkinElmer, Massachusetts, USA) and quantified using the *Qubit dsDNA Assay* kit (Thermo Fisher Scientific, Massachusetts, USA).

Libraries were prepared using either the *Agilent SureSelectXT Human All Exon V6* + *UTR* kit (Agilent Technologies, Santa Clara, CA, USA) (*n* = 381) or the *IDT xGen Gene Capture Pool* (Integrated DNA Technologies, Coralville, Iowa, USA) (*n* = 219). Libraries were sequenced with 150 bp paired-end reads on Illumina NovaSeq6000 or NextSeq550 platforms, (Illumina Inc., San Diego, CA, USA), achieving mean read depths of 53× and 629× across targeted exons, respectively.

Copy number variants (CNVs) were confirmed using the SALSA® digitalMLPA™ Probemix D001-C1 Hereditary Cancer Panel 1 (MRC Holland, Amsterdam, The Netherlands).

### 2.3 Bioinformatics analysis

Targeted analysis was performed using a 19-gene cancer-predisposition panel, including homologous recombination genes (*ATM*, *BRCA1*, *BRCA2*, *BRIP1*, *CHEK2*, *PALB2*, *RAD51C,* and *RAD51D*), mismatch repair genes (*MLH1*, *MSH2*, *MSH6,* and *PMS2)* and additional cancer-associated genes (*CDH1*, *EPCAM [3’ deletions]*, *HOXB13, NBN, PTEN*, *TP53* and *STK11*).

Data were processed using DRAGEN v.4.4.4, Picard 3.4.0, mosdepth 0.3.11, bedtools v2.31.1, Haplogrep v3.2.2., SnpEff and ANNOVAR. GRCh38 build was used as the reference genome.

### 2.4 Variant prioritization and classification

Exonic and flanking-region variants with a minor allele frequency <1% were prioritized and classified according to American College of Medical Genetics and Genomics and the Association for Molecular Pathology (ACMG-AMP) Standards and Guidelines [13], and general ClinGen recommendations [14]. Gene-specific recommendations were also applied (Supplementary Table 2)*.*

### 2.5 Oncologic outcomes

To evaluate the association between P/LP variant carrier status and oncologic outcomes, the following endpoints were defined. The primary endpoint was overall survival (OS), defined as time from the end of radiotherapy to death from any cause. Secondary endpoints included BCR (defined as a PSA increase of ≥2 ng/ml over nadir), distant metastasis (DM), PCSM, and development of a second primary cancer. All endpoints were assessed from the completion of radiotherapy to the date of event occurrence or last follow-up, at which time patients were censored.

### 2.6 Statistical analysis

Descriptive statistics were used to summarize the data. Categorical variables were reported as frequencies, whereas continuous variables were summarized using the median and IQR.

Fisher’s exact test was used to compare the prevalence of P/LP variants in CSGs between patients with high-risk prostate cancer and non-cancer controls cohorts, and to assess differences in categorical variables between carrier and non-carrier groups. The Mann-Whitney U test was used for comparison of non-normally distributed continuous variables (eg, age and PSA level at diagnosis).

The Kaplan-Meier method was used to estimate OS, with differences between groups compared with the log-rank test. Multivariable Cox proportional hazards regression models were fitted to evaluate P/LP variant carrier status as an independent predictor of OS. For secondary endpoints, cumulative incidence functions were estimated using competing risks methodology. Between-group differences were assessed using Gray’s test, and the independent effect of P/LP variants on each endpoint was evaluated using Fine-Gray subdistribution hazard models. All models were adjusted for the following covariates: age (continuous), PSA level (continuous), P/LP variant carrier status (non-carrier vs carrier), Gleason score (<8 vs ≥8), tumor stage (T1–T2 vs T3–T4), and nodal involvement (N0 vs N1). Additional treatment-related variables and comorbidities were explored as potential covariates through stepwise selection in sensitivity analyses and subsequently incorporated in the models (Supplementary Table 3). A complete-case approach was used in the multivariable models, whereby individuals with missing values in any covariates were excluded from the corresponding analysis. Missing data frequencies are reported in Supplementary Table 1.

All statistical tests were two-sided, with a *p* value <0.05 considered statistically significant. Gene-level comparisons between patients and non-cancer controls were adjusted using Bonferroni correction. All analyses were performed using R (version 4.2.2).

## 3 Results

A total of 600 patients meeting EAU high-risk criteria were included. The median age at diagnosis was 70 yr. Clinicopathological characteristics and treatments are summarized in Table 1.

**Table 1:** Summary of cohort characteristics

Clinical variable	Patients (*N* = 600)
**Age of diagnosis (yr)**	***n* (%)**
Median	70
Quartiles	63–75
Unknown	1 (0.20)
**Ethnicity**	
European ancestry	600 (100)
**PSA at diagnosis (ng/ml)**	
Median	14
Quartiles	8–28
Unknown	37 (6.2)
**Gleason score at diagnosis**	
≤6	92 (15)
7	208 (35)
≥8	294 (49)
Unknown	6 (1.0)
**Primary tumor stage at diagnosis**	
T1	49 (8.2)
T2	251 (42)
T3	246 (41)
T4	13 (2.2)
Unknown	41 (6.8)
**Lymph node involvement at diagnosis**	
N0	435 (73)
N1	91 (15)
Unknown	74 (12)
**Personal history of other primary cancer**	48 (8.0)
**N° of reported FDR/SDR with prostate cancer**	
0	509 (85)
1	60 (10)
2	21 (3.5)
≥3	10 (1.7)
**N° of reported FDR/SDR with breast/ovarian cancer**	
0	563 (94)
1	28 (4.7)
≥2	9 (1.5)
**N° of reported FDR/SDR with CCR/endometrial cancer**	
0	563 (94)
1	30 (5.0)
≥2	7 (1.2)
**N° of reported FDR/SDR with pancreatic cancer**	
0	588 (98)
1	11 (1.8)
≥2	1 (0.20)
**Hormone therapy**	
Yes	546 (91)
No	42 (7.0)
Unknown	12 (2.0)
**Radiotherapy**	560 (93)
**Prostatectomy**	146 (24)

PSA = prostate-specific antigen; FDR = first degree relative; SDR = second degree relative; CCR = colorectal.

### 3.1 Prevalence of P/LP variants in CSGs

P/LP variants were identified in 48 of 600 patients (8.0%). One patient carried pathogenic variants in both *BRCA2* and *CHEK2*. *CHEK2, ATM,* and *BRCA2* were the most frequently altered genes, with P/LP variants observed in 2.3%, 1.8%, and 1.3% of patients, respectively. The remaining P/LP variants were identified in *BRCA1*, *NBN*, *PALB2*, *BRIP1*, *PMS2* and *RAD51D* (Supplementary Table 4). Variants were predominantly single nucleotide variants or small insertions/deletions (indels), with CNVs or structural variants identified in two patients. Two recurrent pathogenic variants were observed: the *CHEK2* variant c.349A>G (*n* = 8) and the *ATM* variant c.3577-1G>C (*n* = 4).

A total of 92 variants of uncertain significance were detected in 120 patients (20%) and were distributed across most analyzed genes, except for *EPCAM* (only deletions considered) and *PTEN* (Supplementary Table 5).

Gene-level analyses comparing P/LP variant prevalence in CSGs between patients with high-risk prostate cancer and reference control groups showed a significantly higher prevalence of P/LP variants in *CHEK2*, *ATM*, and *BRCA2* after Bonferroni correction (Table 2).

**Table 2:** Germline pathogenic and likely pathogenic variants in patients with prostate cancer compared with non-cancer controls

	*N° of pathogenic and likely pathogenic variants*									
	RADIOGEN (*N*_alleles_ = 1200)		Internal cohort (*N*_alleles_ = 21 154)		gnomAD^a^ (*N*avg = 102 570)		RADIOGEN vs Internal cohort		RADIOGEN vs gnomAD^a^	
Gene	*n*	%	*n*	%	*n*	%	Odds Ratio (95% CI)	*p*-value	Odds Ratio (95% CI)	*p*-value
***CHEK2***	14	1.2	79	0.37	93	0.091	3.15 (1.64–5.62)	0.004	13.01 (6.83–23.01)	<0.001
***ATM***	11	0.92	43	0.20	215	0.21	4.54 (2.11–8.98)	0.001	4.40 (2.16–8.07)	<0.001
***BRCA2***	8	0.67	40	0.19	153	0.15	3.54 (1.43–7.69)	0.034	4.49 (1.90–9.11)	0.006
***BRCA1***	5	0.42	27	0.13	109	0.11	3.27 (0.98–8.64)	0.2	3.93 (1.25–9.49)	0.096
***NBN***	3	0.25	6	0.028	35	0.034	8.83 (1.43–41.42)	0.091	7.34 (1.44–23.35)	0.087
***PALB2***	3	0.25	16	0.075	79	0.077	3.31 (0.62–11.59)	0.7	3.25 (0.66–9.88)	0.6
***BRIP1***	2	0.17	20	0.095	70	0.068	1.76 (0.20–7.28)	>0.9	2.44 (0.29–9.19)	>0.9
***PMS2***	2	0.17	7	0.033	79	0.077	5.04 (0.51–26.52)	0.7	2.17 (0.26–8.10)	>0.9
***RAD51D***	1	0.083	19	0.090	19	0.019	0.93 (0.02–5.85)	>0.9	4.50 (0.11–28.37)	>0.9

*All p-values were Bonferroni corrected*.

N_alleles_ = Total alleles/n = Allele count; Navg = Average number of alleles.

a Non-Finnish European (non-cancer).

### 3.2 Clinical variables and carrier status

Germline P/LP carrier status was significantly associated with younger age at diagnosis and with a family history of breast/ovarian cancer, pancreatic cancer, and any cancer. When stratified by gene, *CHEK2* was the only gene for which these family history associations remained statistically significant, including an additional association with a family history of prostate cancer (Table 3).

**Table 3:** Association of pathogenic and likely pathogenic variant carrier status with clinical and pathological variables at diagnosis

	Non-carriers	All P/LP carriers		*CHEK2* carriers		*ATM* carriers		*BRCA2* carriers	
	Total (*n* = 552)	*n* = 48	*p-*value	*n* = 14	*p*-value	*n* = 11	*p*-value	*n* = 8	*p*-value
**Age (yr)**									
Median	70	68	0.042	69	0.3	68	0.3	62	0.057
Quartiles	63–75	62–72		66–72		60–73		60–69	
Unknown	1	0		0		0		0	
**PSA (ng/ml)**									
Median	14	11	0.7	8.8	0.4	11	0.9	21	0.4
Quartiles	7.9–28	9.1–21		7.9–14		10–24		14–26	
Unknown	34	3		1		1		1	
**Gleason score**									
<8	274 (50)	26 (54)	0.7	11 (79)	0.055	5 (45)	0.8	3 (38)	0.7
≥8	272 (49)	22 (46)		3 (21)		6 (55)		5 (63)	
Unknown	6 (1.1)	0		0		0		0	
**Primary tumor stage**									
T1-T2	275 (50)	25 (52)	>0.9	7 (50)	>0.9	5 (45)	0.8	3 (38)	0.7
T3-T4	236 (43)	21 (44)		5 (36)		6 (55)		4 (50)	
Unknown	41 (7.4)	2 (4.2)		2 (14)		0		1 (13)	
**Lymph node involvement**									
N0	402 (73)	33 (69)	0.3	11 (79)	0.7	7 (64)	0.7	6 (75)	>0.9
N1	80 (14)	10 (21)		1 (7.1)		2 (18)		1 (13)	
Unknown	70 (13)	5 (10)		2 (14)		2 (18)		1 (13)	
**Family history of cancer**									
Prostate	82 (15)	9 (19)	0.5	5 (36)	0.049	3 (27)	0.2	2 (25)	0.3
Breast/Ovarian	30 (5.4)	7 (15)	0.022	4 (29)	0.007	1 (9.1)	0.5	2 (25)	0.071
Colorectal/Endometrial	32 (5.8)	5 (10)	0.2	1 (7.1)	0.6	2 (18)	0.14	2 (25)	0.080
Pancreatic	8 (1.4)	4 (8.3)	0.011	2 (14)	0.023	1 (9.1)	0.16	1 (13)	0.12
Any^a^	143 (26)	20 (42)	0.027	8 (57)	0.014	5 (45)	0.17	3 (38)	0.4

PSA = Prostate-Specific Antigen; P/LP = Pathogenic and likely pathogenic.

a Any includes: prostate, breast/ovarian, colorectal/endometrial, pancreatic and any other types of cancer.

### 3.3 Oncologic outcomes

Survival outcomes were analyzed in a predefined subset of 390 patients primarily treated with radical radiotherapy, focusing on carriers of P/LP variants in the most frequently altered genes: *CHEK2* (*n* = 9)*, ATM* (*n* = 7)*, BRCA1* (*n* = 4) and *BRCA2* (*n* = 3). *BRCA1* and *BRCA2* carriers were grouped due to limited sample sizes. Non-carriers were defined as patients without P/LP variants in any gene of the panel. Baseline clinicopathological characteristics, comorbidities, and treatment parameters are presented in Supplementary Table 1.

#### 3.3.1 OS

**Fig. 1:**
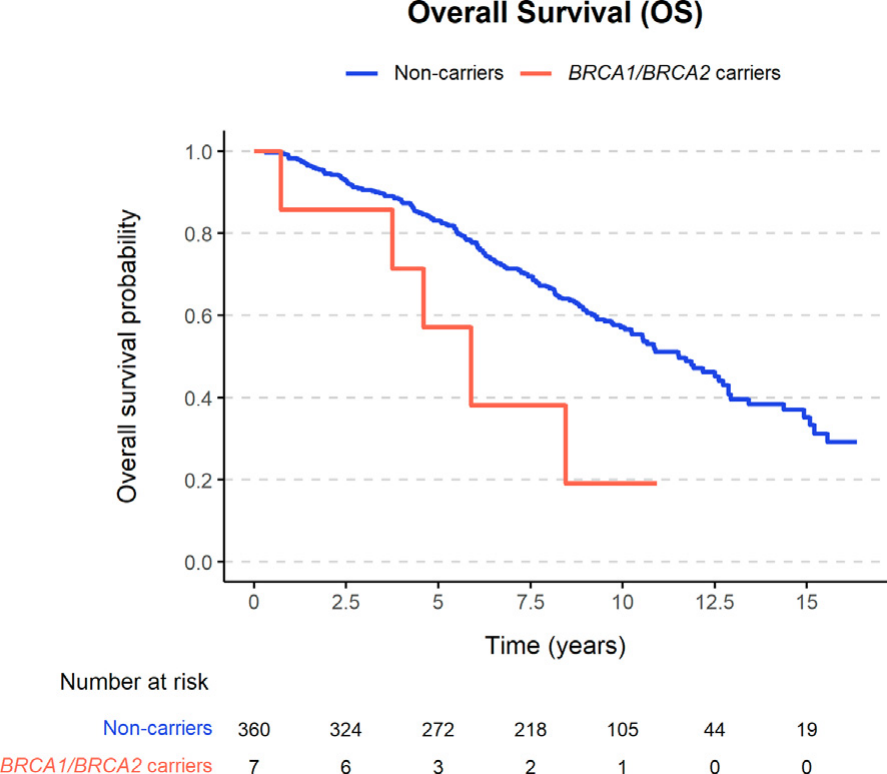
Kaplan-Meier curves for overall survival stratified by *BRCA1*/*BRCA2* carrier status.

Patients with *BRCA1* or *BRCA2* P/LP variants showed poorer OS compared with non-carriers. The OS rates at 3, 5, and 10 yr were 86%, 57%, and 19% for carriers versus 91%, 83%, and 57% for non-carriers (log-rank *p* = 0.021) (Supplementary Table 6; Fig. 1). In multivariable analysis, *BRCA1* and *BRCA2* variant status remained independently associated with OS (hazard ratio [HR] = 2.73; 95% confidence interval [CI], 1.11–6.71; *p* = 0.028). No statistically significant differences in OS were observed for *ATM* or *CHEK2* carriers (Supplementary Fig. 1).

#### 3.3.2 Biochemical recurrence, distant metastasis, and prostate cancer–specific mortality

**Fig. 2:**
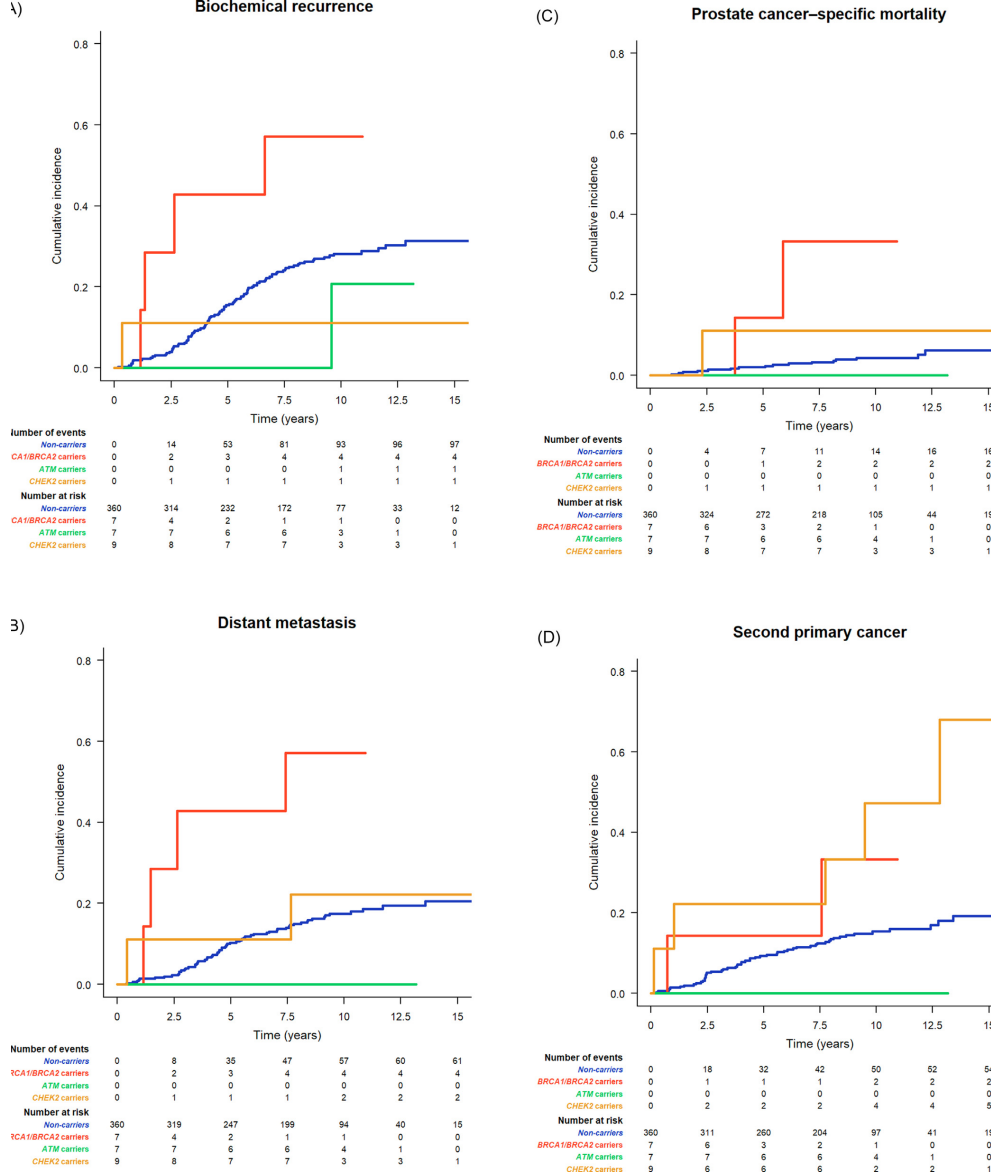
Cumulative incidence of secondary endpoints stratified by P/LP variant carrier status. The *p* values in parentheses represent Gray’s test comparing P/LP carriers of each gene versus non-carriers. (A) Cumulative incidence of biochemical recurrence among *CHEK2* (*p* = 0.3), *ATM* (*p* = 0.4), and *BRCA1*/*BRCA2* (*p* = 0.048) carriers. (B) Cumulative incidence of distant metastasis among *CHEK2* (*p* = 0.7), *ATM* (*p* = 0.2), and *BRCA1*/*BRCA2* (*p* = 0.004) carriers. (C) Cumulative incidence of prostate cancer–specific mortality among *CHEK2* (*p* = 0.4), *ATM* (*p* = 0.6), and *BRCA1*/*BRCA2* (*p* = 0.001) carriers. (D) Cumulative incidence of second primary cancers among *CHEK2* (*p* = 0.003), *ATM* (*p* = 0.3), and *BRCA1*/*BRCA2* (*p* = 0.3) carriers. P/LP = pathogenic and likely pathogenic.

*BRCA1* and *BRCA2* carriers had a significantly higher cumulative incidence of BCR, DM, and PCSM (Fig. 2A–C; Supplementary Table 6). In multivariable analyses, *BRCA1* and *BRCA2* mutation status remained independently associated with all three endpoints (Supplementary Table 7). In contrast, the cumulative incidence of these outcomes in carriers of *ATM* or *CHEK2* mutations did not differ significantly from that observed in non-carriers. No metastatic events or prostate cancer–specific deaths were observed among *ATM* carriers during follow-up.

#### 3.3.3 Second primary cancer incidence after prostate cancer

*CHEK2* P/LP variant carriers had a significantly higher cumulative incidence of second primary cancers compared with non-carriers (10-yr cumulative incidence: 47% vs 15%; *p* = 0.003) (Fig. 2D; Supplementary Table 6). The increased incidence encompassed a broad spectrum of malignancies, including appendiceal, bladder and pancreatic cancers, and basal cell carcinoma, brain tumors, leukemia, and sarcoma. Consistently, in multivariable analysis, *CHEK2* P/LP variant carrier status remained independently associated with second primary malignancy risk (Supplementary Table 7).

## 4 Discussion

Although germline genetic testing is increasingly being integrated into prostate cancer management, evidence specifically addressing high-risk non-metastatic disease remains limited. In our cohort of 600 high-risk patients, 8.0% carried a P/LP variant in a CSG. This prevalence exceeds that reported in unselected localized cohorts [15]; however, remains lower than rates observed in metastatic populations [6]. Notably, *CHEK2*, *ATM*, and *BRCA2* were enriched in our high-risk population. These genes have consistently been identified as prostate cancer–predisposition genes across multiple independent studies [16], [17], [18], [19], [20]. Of note, to mitigate the inherent limitations of public databases as reference populations, including the lack of cancer-free status, differences in ancestry, and variability in sequencing and variant-calling pipelines, comparisons were complemented with an internal control cohort sharing similar ancestry background and processed using comparable analytical pipelines, thereby providing a more robust reference framework and supporting the validity of our findings. Our results support the relevance of these genes in the high-risk localized setting and highlight the potential clinical utility of germline testing in this patient population.

In our cohort, *BRCA1* and *BRCA2* carriers exhibited markedly worse OS and higher rates of BR, DM, and PCSM compared with non-carriers. Multivariable analyses confirmed the association of *BRCA1* and *BRCA2* alterations with poorer outcomes, supporting their potential role as biomarkers of aggressive disease, as previously described [19], [21]. Comparable adverse outcomes have also been observed in *BRCA1* and *BRCA2* carriers undergoing radical prostatectomy [22]. These findings raise the question of whether PARP inhibitors could be explored earlier in curative-intent treatment strategies. Although PARP inhibitors have demonstrated survival benefits in castration-resistant metastatic prostate cancer [23], their role in localized or locally advanced disease has not yet been established. In addition to their systemic activity, preclinical data suggest that PARP inhibition may enhance tumor radiosensitivity [24], providing a rationale for further investigation in combination with definitive radiotherapy.

*CHEK2* carriers also exhibited distinctive clinical and genetic features in our cohort. They more frequently reported relatives affected by prostate, breast/ovarian, pancreatic, or other cancers, and displayed a higher cumulative incidence of second primary malignancies with a heterogeneous tumor landscape. This pattern further supports *CHEK2’s* role as a multiorgan CSG [25], associated with malignancies beyond the hereditary breast and ovarian cancer spectrum [26], [27], [28]. Nevertheless, moderate risk estimates, the variability in tumor types, and the relatively high proportion of unaffected carriers pose challenges for implementing gene-specific surveillance strategies, warranting further investigation in prospective studies.

*ATM* carriers demonstrated clinical outcomes of potential relevance in the radiotherapeutic setting. Radiosensitivity is a hallmark of ataxia–telangiectasia, a rare autosomal recessive disorder caused by biallelic germline inactivation of *ATM*. Previous studies have suggested that *ATM* alterations may enhance tumor control following radiotherapy. For instance, Pitter et al [29] reported that both monoallelic (germline or somatic) alterations and biallelic tumor inactivation of *ATM* were associated with reduced progression across multiple irradiated tumor types. Similarly, Ma et al [30] described exceptional responses in eight patients with diverse malignancies harboring somatic *ATM* alterations. However, short follow-up durations and heterogeneity in patient selection have limited the robustness of these conclusions. A notable strength of our study is the stringent selection of the cohort and the long follow-up period. With a median of 10.6 yr among *ATM* carriers, none of them experienced metastatic progression or PCSM. These results are consistent with increased radiosensitivity in *ATM*-deficient tumors, supporting the potential role of *ATM* status as a clinically relevant biomarker in the radiotherapeutic setting.

Taken together, the four genes exhibit distinct biological and clinical profiles. *BRCA1* and *BRCA2* are associated with aggressive disease and poor prognosis, *CHEK2* with multiorgan cancer susceptibility and risk of second malignancies, and *ATM* with potential radiosensitivity. This heterogeneity highlights the potential of germline genetic profiling to support risk-adapted surveillance and management strategies, contributing to precision oncology in high-risk prostate cancer. However, some limitations should be acknowledged. Outcome analyses were restricted to patients treated with radiotherapy (*n* = 390), who may not fully represent the overall cohort. Treatment approaches and comorbidities were accounted for in multivariable models; however, limitations associated with stepwise covariate selection and residual confounding cannot be dismissed. Additionally, performance status was unavailable and could not be included. Beyond this, the generalizability of these results may be limited. The relatively small number of P/LP carriers per gene, although reflecting the rarity of these variants in clinical practice, may be insufficient to yield robust statistical conclusions. Single-institution design further tempers the interpretation of these findings, given the inherent variability in referral patterns, clinical management, and population characteristics across centers. For these reasons, these gene-specific observations warrant cautious interpretation and prospective validation in larger, independent cohorts before they can be translated into clinical practice. Nevertheless, the long follow-up and comprehensive genetic characterization strengthen the validity of our findings.

## 5 Conclusions

Our study provides preliminary insights into the potential clinical relevance of germline genetic testing in high-risk prostate cancer. We found that 8.0% of patients with high-risk prostate cancer carry P/LP variants in CSGs, with *CHEK2, ATM* and *BRCA2* emerging as the most frequently altered genes. *BRCA1* and *BRCA2* carriers were associated with worse oncologic outcomes, supporting their role as potential prognostic biomarkers and highlighting the need for risk-adapted therapeutic strategies. *CHEK2* and *ATM* carriers demonstrated distinct clinical and biological profiles, suggesting opportunities for personalized surveillance and treatment approaches. Nonetheless, given the retrospective design of this study and the limited sample size, these findings should be interpreted as hypothesis-generating. Prospective, larger-scale studies are warranted to validate these associations and define the clinical utility of germline testing in guiding management of high-risk prostate cancer.

  ***Author contributions:*** Ana Vega had full access to all the data in the study and takes responsibility for the integrity of the data and the accuracy of the data analysis.

  *Study concept and design*: Vega, Galego-Carro.

*Acquisition of data*: Blanco-Pérez, Aguado-Barrera, Amigo, Galego-Carro, Calvo-Crespo, Peleteiro-Higuero, Carballo-Castro, Taboada-Valladares, Gómez-Caamaño.

*Analysis and interpretation of data*: Galego-Carro, Santamariña, Coedo-Costa, Vega.

*Drafting of the manuscript*: Galego-Carro, Vega.

*Critical revision of the manuscript for important intellectual content*: All authors.

*Statistical analysis*: Galego-Carro, Aguado-Barrera, Vega.

*Obtaining funding*: Vega.

*Administrative, technical, or material support*: None.

*Supervision*: Vega.

*Other*: None.

  ***Financial disclosures:*** Ana Vega certifies that all conflicts of interest, including specific financial interests and relationships and affiliations relevant to the subject matter or materials discussed in the manuscript (eg, employment/affiliation, grants or funding, consultancies, honoraria, stock ownership or options, expert testimony, royalties, or patents filed, received, or pending), are the following: None.

  ***Funding/Support and role of the sponsor:*** The name of the organization or organizations which had a role in sponsoring the data and material in the study are also listed below:

Instituto de Salud Carlos III, Xunta de Galicia, Asociación Española Contra el Cáncer.

  ***Declaration of Generative AI and AI-assisted technologies in the writing process:*** During the preparation of this manuscript, the authors utilized ChatGPT and Claude only to enhance language and grammar. Following the use of this tool, the authors thoroughly reviewed and revised the content as necessary and take full responsibility for the accuracy and integrity of the final content of the publication.

***Acknowledgements:*** A.Vega research is supported by ISCIII funding, an initiative of the Spanish Ministry of Economy and Innovation partially supported by European Regional Development FEDER Funds (PI25/00744, PI22/00589, INT24/00023, DTS24/00083), the Spanish Association Against Cancer AECC (PRYES211091VEGA), and the Autonomous Government of Galicia (Consolidation and structuring programme: IN607B-2025-09). J. Galego-Carro was supported by a Predoctoral Fellowship (Ministerio de Ciencia, Innovación y Universidades: FPU2022/04048). This research project was made possible through the access granted by the Galician Supercomputing Center (CESGA) to its supercomputing infrastructure.

## Appendix A Supplementary material

Supplementary Fig. 1 Kaplan-Meier survival curves for overall survival stratified by carrier status. (A) Comparison between *ATM* P/LP variant carriers and non-carriers showed no statistically significant difference in overall survival (log-rank *p* = 0.4; HR = 0.53, 95% CI: 0.13–2.20, *p* = 0.4). (B) Similarly, no significant difference was observed between *CHEK2* P/LP variant carriers and non-carriers (log-rank *p* = 0.3; HR = 0.84, 95% CI: 0.26–2.69, *p* = 0.8). P/LP = pathogenic and likely pathogenic; HR = hazard ratio; CI = confidence interval.

Supplementary Tables **Supplementary Table 1.** Clinicopathological characteristics and treatment parameters of patients included in the oncologic outcome analysis. **Supplementary Table 2.** Variant classification guidelines used. **Supplementary Table 3.** Results of stepwise covariate selection for multivariable analyses. **Supplementary Table 4.** Pathogenic and likely pathogenic variants detected in the cohort. **Supplementary Table 5.** Variants of uncertain significance detected in the cohort. **Supplementary Table 6.** 3-, 5- and 10-yr survival and event rates (%) for all endpoints. **Supplementary Table 7.** Multivariable analysis of secondary endpoints.
