# Supplementary material for: Clinical Impact of Germline Pathogenic Variants in High-risk Prostate Cancer Treated with Radiotherapy
Source: Eur Urol Open Sci. 2026 Jul 27;91:49–58. doi: 10.1016/j.euros.2026.07.002 (PMC13446358; doi:10.1016/j.euros.2026.07.002)
Supplement: Supplementary file 1 [file mmc1.docx]

**B)**

**A)**

**Supplementary Figure 1.** Kaplan–Meier survival curves for overall survival stratified by carrier status. (A) Comparison between *ATM* P/LP variant carriers and non-carriers showed no statistically significant difference in overall survival (log-rank *p* = 0.4; HR = 0.53, 95% CI: 0.13–2.20, *p* = 0.4). (B) Similarly, no significant difference was observed between *CHEK2* P/LP variant carriers and non-carriers (log-rank *p* = 0.3; HR = 0.84, 95% CI: 0.26–2.69, *p* = 0.8).
